# Influence of Nonpolar Substances on the Extraction Efficiency of Six Alkaloids in Zoagumhwan Investigated by Ultra Performance Liquid Chromatography and Photodiode Array Detection

**DOI:** 10.3390/molecules171213844

**Published:** 2012-11-22

**Authors:** Yanling Zhao, Lei Jia, Hongbo Yang, Jiabo Wang, Ping Zhang, Ruisheng Li, Man Gong, Shengqiang Luo, Shijing Liu, Xiaohe Xiao

**Affiliations:** 1 China Military Institute of Chinese Medicine, 302 Hospital of People’s Liberation Army, 100, the 4th Ring Road, Beijing 100039, China; 2 School of Chinese Medicine, The University of Hong Kong, Estates Building, 10 Sassoon Road, 999077, Hong Kong; 3 Yunnan Institute of Materia Medica, 22, Lengshuitang, Xishan District, Kunming, Yunnan 650111, China; 4 Department of Integrative Medical Center, 302 Hospital of the People’s Liberation Army 100, 4th Ring Road, Beijing 100039, China; 5 Animal Laboratory Center, 302 Hospital of the People’s Liberation Army, 100, 4th Ring Road, Beijing 100039, China

**Keywords:** alkaloids, nonpolar substances, Zoagumhwan, UPLC-PDA, extraction efficiency

## Abstract

A reverse phase ultra performance liquid chromatography and photodiode array (UPLC-PDA) detection method was established for the determination of six alkaloids in Zoagumhwan (ZGW), and further for investigating the influence of nonpolar substances on the extraction efficiency of these alkaloids. The method was based on a BEH C_18_ (50 mm × 2.1 mm, 1.7 μm) column and mobile phase of aqueous phosphoric acid and acetonitrile including 0.05% buffer solution under gradient elution. ZGW samples of ZGW I, II, III and IV were obtained and prepared by pre-processing the crude materials of *Coptidis rhizoma* and *Evodiae fructus* using four technologies, namely direct water decoction, removal of nonpolar substances in *Evodiae fructus* by supercritical fluid extraction (SFE), removal of nonpolar substances in ZGW by SFE and removal of nonpolar substances in ZGW by steam distillation. The developed and validated UPLC-PDA method was precise, accurate and sensitive enough based on the facts that the six alkaloids showed good regression (*r *> 0.9998), the limit of detections and quantifications for six alkaloids were less than 28.8 and 94.5 ng/mL, respectively, and the recovery was in the range of 98.56%–103.24%. The sequence of the total contents of six alkaloids in these samples was ZGW II > ZGW IV > ZGW III > ZGW I. ZGW II, in which nonpolar substances, including essential oils, were firstly removed from *Evodiae fructus* by SFE, had the highest content of the total alkaloids, indicating that extraction efficiency of the total alkaloids could be remarkably increased after *Evodiae fructus* being extracted by SFE.

## 1. Introduction

Traditional Chinese medicines (TCMs) and their compound preparations have become more and more popular around the World during the last decade for their therapeutic effects which are complementary to Western medicines, and their capability to deal with many essential problems that have not yet been solved by current medicinal practices [1]. Zoagumhwan (ZGW) is a well-known compound preparation composed of *Coptidis rhizoma* and *Evodiae fructus *at the ratio of 6:1 (w/w), and has been used for more than 1,000 years. It is one of the simplest compound preparations but retains the basic therapeutic features [2].

**Figure 1:**
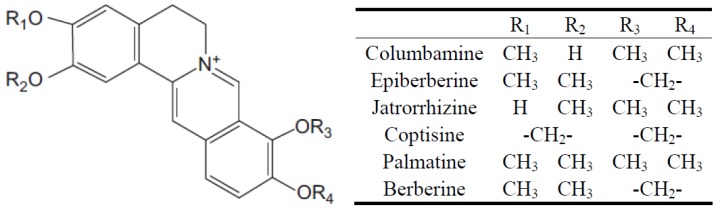
Structures of six tested alkaloids.

Today, ZGW has been officially listed in the Chinese Pharmacopoeia [3] for its satisfactory effects of healing hypochondric and costal pain, stomach ache, acid reflux and nausea. It is reported [4,5,6,7] that the major active components of Zoagumhwan are alkaloids, namely columbamine, epiberberine, jatrorrhizine, coptisine, palmatine and berberine, as shown in Figure 1.

To date, many factors shown to affect the extraction efficiency of these alkaloids from Zoagumhwan, such as altering extraction solution [8], decoction conditions, sequence of decoction [9], *etc.*, have been investigated. However, it is well-known that *Evodiae fructus*, one of the components in Zoagumhwan, contains a volatile fraction, and monoterpenes and sesquiterpenes are the main volatile components in the volatile sample of *Evodia* according to gas chromatography-mass spectrometry (GC-MS) analysis [10,11,12]. Nevertheless, the influence of the nonpolar substances, including essential oils and other substances, on the extraction efficiency of the alkaloids was neglected in previous studies. In decoction, it is very easy that volatile components are freed from the herb. A small amount of them can be dissolved in the extraction solution, and this might affect the extraction of alkaloids from Zoagumhwan. Therefore, it is significant to investigate the influence of nonpolar substances on extraction efficiency of the main alkaloids.

Supercritical fluid extraction (SFE) and steam distillation are the most commonly utilized methods to extract nonpolar substances from medicinal and edible plant foods. In this study, the two methods were employed to remove the nonpolar fraction. Four different protocols, including direct water decoction, removal of nonpolar substances in *Evodiae fructus* by SFE, removal of nonpolar substances in Zoagumhwan by SFE and removal of nonpolar substances in Zoagumhwan by steam distillation were used to pre-process crude materials of *Coptidis rhizoma* and *Evodiae fructus *for obtaining different Zoagumhwan samples. Then, a rapid ultra performance liquid chromatography and photodiode array (UPLC-PDA) detection method was developed for the simultaneous determination of the six alkaloids in these extracts.

## 2. Results and Discussion

### 2.1. Development of the UPLC Method

In the course of developing the rapid UPLC method, the basic chromatographic conditions such as mobile phase, flow rate, elution conditions, column temperature and detection wavelength were taken into consideration.

Mobile phase was a major factor for the good separation of the six tested alkaloids. Firstly, methanol, which was found to be less selective in the separation of the six alkaloids than acetonitrile, was discarded. According to the reports [13,14], the composition of acetonitrile and 20 mM ammonium acetate, which was adjusted to pH 10 by ammonia water, was investigated first. The results showed that the alkaloids could be separated well, but serious tailing was observed. Therefore, in order to improve the tailing, an acid solvent was considered according to the acid-base equilibrium theory. Choosing acetonitrile-0.5% (*v/v*) acetic acid could give an acceptable performance, but the separation between columbamine, jatrorrhizine, epiberberine and coptisine was not satisfactory. Finally, a more satisfactory chromatographic separation was acquired through the combination of acetonitrile and 0.05% (*v/v*) phosphoric acid water solution. A gradient elution procedure as shown in Table 1 was performed in order to decrease the total run time. 

**Table 1:** UPLC gradient elution procedure.

Time (min)	Flow rate (mL/min)	(A) 0.05% phosphoric acid water	(B) Acetonitrile
0.0	0.40	85%	15%
1.0	0.40	85%	15%
1.5	0.28	80%	20%
4.5	0.28	80%	20%
5.0	0.40	75%	25%
8.0	0.40	75%	25%

Attempts to further improve the chromatographic performance, the flow rate and the column temperature were adequately investigated. The experiments showed that the influence of column temperature was very remarkable on the separation of COL, JAT, EPI and COP. When the column temperature was raised from 15 °C to 40 °C, resolution was notablely degraded and the number of theoretical plates was raised notably. Finally, it was preferred to the column temperature of 22 °C. Meanwhile, with a reduced flow rate, the tailing factor of the six alkaloids was increased.

**Figure 2:**
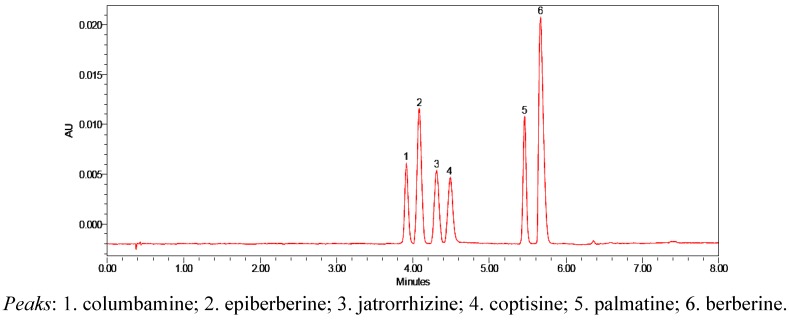
UPLC-PDA chromatograms of mixed standard solution.

Three different UPLC-PDA detection wavelengths of 220, 270 and 340 nm were monitored and compared. 270 nm was finally selected for the lowest baseline noise for berberine, which was observed to be the best detection wavelength for all the subsequent experiments. Under the optimized UPLC conditions, the satisfactory and rapid separation of the six tested alkaloids shown in Figure 2 was achieved in 6 min with a back pressure of 7,800 psi.

### 2.2. Validation of the UPLC Method

The UPLC method was validated in terms of linearity, precision and accuracy according to ICH guidelines [15].

#### 2.2.1. Calibration Curves

Methanol stock solutions containing six reference compounds were prepared and diluted to appropriate concentrations for the construction of calibration curves. At least six concentrations of the solution were analyzed in triplicate, and then the calibration curves were constructed by plotting the peak areas (*y*) *versus* the concentration (*x*) of each tested alkaloid. The results are shown in Table 2. The linearity of the calibration curves was verified by a correlation study and the correlation coefficients (*r*) were all better than 0.9998 within the test ranges.

#### 2.2.2. Sensitivity

The methanol stock solution containing the six reference compounds was diluted to a series of appropriate concentrations with the same solvent, and an aliquot of the diluted solutions was injected into the UPLC system for PDA analysis. The limits of detection (LODs) and quantification (LOQs) under the present chromatographic conditions were determined at a signal-to-noise ratio (S/N) of about 3 and 10, respectively. Table 2 showed the data of LOD and LOQ for each investigated compound. The confidence LODs and LOQs for the six alkaloids were 25.8–31.8 and 85.0–104 ng/mL with 95% confidence level, respectively.

**Table 2:** Linear regression data, LOD and LOQ of the six investigated alkaloids.

Analyte	Resolution ^a^	Linear regression data			LOD (ng/mL)	LOQ (ng/mL)
		Regression equation ^b^	Test range (μg/mL)	*r*		
Columbamine	—	*y *= 19154119*x* + 867.02	0.19–31.20	0.9998	16.3	55.4
Epiberberine	2.02	*y* = 19767219*x* − 628.2	0.25–38.10	1.0000	25.8	92.9
Jateorrhizine	3.02	*y* = 17377169*x* + 1454.2	0.17–27.50	0.9998	21.5	72.0
Coptisine	1.53	*y* = 18125088*x* − 91.1	0.38–65.00	0.9999	22.3	76.3
Palmatine	10.61	*y* = 10386051*x* − 699.2	0.75–12.00	0.9999	19.6	64.3
Berberine	2.47	*y* = 12063621*x* + 2611.3	1.44–144.00	0.9999	28.8	94.5

^a^ Resolution of the peaks of six alkaloids in UPLC profile was calculated using the Waters Empower 2 software; ^b^ *y*, peak area, *x*, concentration of each tested alkaloid.

#### 2.2.3. Precision

Intra- and inter-day variations were chosen to determine the precision of the developed method. For the intra-day variability test, six replicates of the mixed standards solution were analyzed within 1 day, while for the inter-day variability test, the solution was examined in duplicate for three consecutive days. For every calibration curve, the calibration concentrations were back-calculated from the peak area of the standard substances. The deviation from the nominal concentration was defined as accuracy. As shown in Table 3, variations, which were expressed by the intra- and inter-day relative standard deviations (RSD), were less than 0.9 and 1.2%, respectively. 

#### 2.2.4. Stability

With the temperature of both the laboratory and the control of the column and the auto-sample cell, being 22 °C, samples usually have to be stay at 22 °C for a long time, which should be considered carefully for unstable standard substances. In this study, the stability of the six alkaloids was tested by injecting standard solution at 0, 1, 2, 4, 8, 12 and 18 h at 22 °C. The results showed that all samples were stable under these conditions during the test time period (Table 3). Thus, the quantification of investigated compounds in all samples was preformed within 18 h.

#### 2.2.5. Recovery

The recovery of the method was determined by spiking known amount of mixed standards in a certain amount (75 mg) of extract abtianed by direct water decoction. Three replicates were performed for the test. The mixture was extracted and analyzed using the method mentioned above. The results were calculated and are given in Table 4. The average recoveries of the five alkaloids were 98.56%–103.24%, with RSD values of less than 0.9%.

**Table 3:** Stability of the tested alkaloids, intra- and inter-day precision of the UPLC method.

Analyte	Stability (RSD, %)	Intra-day precision (n = 6)		Inter-day precision (n = 6)	
	22 °C	Accuracy (%) ^a^	RSD (%)	Accuracy (%)	RSD (%)
Columbamine	1.0	100.1	0.8	100.4	1.2
Epiberberine	0.6	97.7	0.3	98.1	0.8
Jateorrhizine	0.8	101.3	0.9	100.8	0.9
Coptisine	0.8	98.0	0.7	98.5	1.0
Palmatine	0.8	99.6	0.6	99.7	1.2
Berberine	0.4	103.3	0.3	102.2	0.4

^a^ Accuracy (%) = 100 × (mean measured concentration/nominal concentration).

**Table 4:** Recoveries for sample spiked with investigated alkaloids (n = 3).

Analyte	Original (μg)	Spiked (μg)	Found ^a^ (μg)	Recovery ^b^ (%)	RSD (%)
Columbamine	200.7	228.2	436.3	103.24	0.9
Epiberberine	288.9	324.5	619.5	101.88	0.8
Jateorrhizine	232.4	244.3	481.6	102.00	0.6
Coptisine	524.0	501.2	1018.0	98.56	0.8
Palmatine	816.8	885.2	1713.2	101.26	0.3
Berberine	2587.0	2474.6	5053.6	99.68	0.2

^a^ Average of triplicates; ^b^ Recovery (%) = 100 × (amount found − original amount)/spiked amount.

All the above results illustrate that the developed UPLC-PDA method was precise, accurate and sensitive enough for simultaneous quantitative determination of the six alkaloids in Zoagumhwan samples.

### 2.3. Analysis of Real Samples

**Figure 3:**
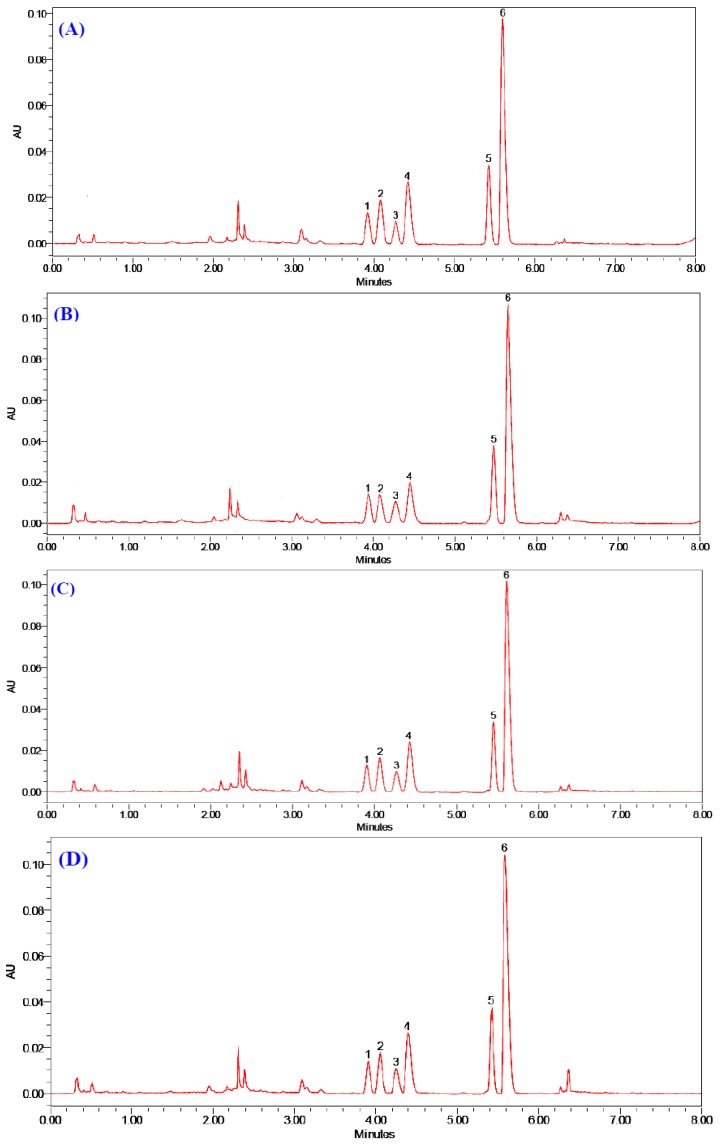
UPLC-PDA chromatograms of (**A**) ZGW I, (**B**) ZGW II, (**C**) ZGW III and (**D**) ZGW IV.

In this study, the contents of six alkaloids in four kinds of Zoagumhwan samples identified as ZGW I, II, III and IV from four different pre-processing methods were determined rapidly and simultaneously with the developed UPLC-PDA method. To reduce experimental error, each sample was extracted and analyzed in triplicate. The typical UPLC-PDA chromatograms of Zoagumhwan samples (ZGW I, II, III and IV) are shown in Figure 3. The target components were identified by comparing their retention times and UV spectra with those presented in the chromatogram of the mixture standard solution (Figure 2). The peak purity of target components from the four samples was verified using a photodiode array detector and found to be satisfactory. It could be seen from Figure 3 that the peak height and peak area of the same component had slight differences, though the number of predominant peaks from the four samples didn’t change. Comparing the six characteristic peaks in the four chromatograms, one could find easily that peak 6 (berberine) is the most important active constituent in Zoagumhwan with the highest peak height. 

In order to further find the differences between the same components in the four Zoagumhwan samples, the contents (mg/g) of the six investigated alkaloids in the four kinds of Zoagumhwan samples (ZGW I, II, III and IV) were determined in triplicate and listed in Table 5.

**Table 5:** Contents (mg/g) of investigated alkaloids in four kinds of Zoagumhwan samples.

Analytes	ZGW I ^a^	ZGW II	ZGW III	ZGW IV
Columbamine	2.81 ^b^ ± 0.08	3.25 ± 0.08	3.01 ± 0.07	3.15 ± 0.09
Epiberberine	3.90 ± 0.07	2.96 ± 0.08	3.37 ± 0.06	3.27 ± 0.08
Jateorrhizine	2.70 ± 0.04	3.44 ± 0.05	2.72 ± 0.04	2.91 ± 0.04
Coptisine	6.85 ± 0.08	5.28 ± 0.10	5.86 ± 0.07	6.25 ± 0.09
Palmatine	11.02 ± 0.11	13.50 ± 0.13	10.69 ± 0.11	11.67 ± 0.09
Berberine	35.86 ± 0.72	41.58 ± 0.77	37.98 ± 0.68	38.58 ± 0.75
Total	63.14 ± 0.65	70.01 ± 0.71	63.63 ± 0.54	65.83 ± 0.69
Increment (%) ^c^	——	10.9	0.8	4.3

^a^ Zoagumhwan sample from four pre-processing methods on crude materials; ^b^ Average of triplicates; ^c^ Increment (%) = 100 × (the contents of total alkaloids in ZGW II, III or IV − ZGW I)/ZGW I.

From the quantitative results, one could find that the contents of total alkaloids in the four samples showed significant differences. The sequence of the contents of total alkaloids in these samples was ZGW II > ZGW IV > ZGW III > ZGW I. ZGW II, in which nonpolar substances were firstly removed from *Evodiae fructus* by SFE-CO_2_, had the highest content of total alkaloids. The results indicated that removal of nonpolar substances from *Evodiae fructus* could remarkably increase the extraction efficiency of the total alkaloids. In addition, the contents of total alkaloids in ZGW III and ZGW IV were lower than that in ZGW II. The reasons might be that parts of alkaloids in ZGW III were extracted by SFE-CO_2_, and the extraction efficiency of alkaloids in ZGW IV was decreased by the remaining nonpolar substances. The increment, which was calculated by the formula: increment (%) = 100 × (the contents of total alkaloids in ZGW II, III or IV- ZGW I)/ZGW I, of ZGW II, III and IV were 10.9%, 0.8% and 4.3%, respectively. 

**Figure 4:**
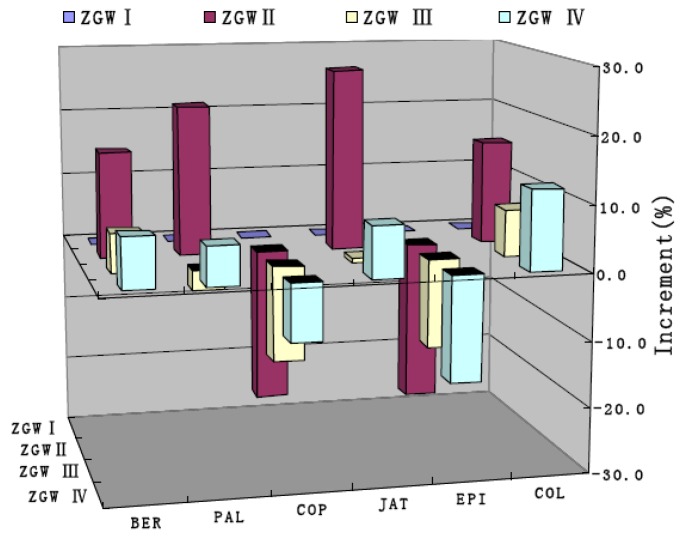
Influence of essential oil on the content of six alkaloids in Zoagumhwan samples.

On the other hand, the content of berberine was still the highest among the six major constituents in the samples, and the content of each alkaloid was influenced by nonpolar substances, but from Figure 4, it could be found that compared with the ZGW I sample, the content of columbamine and berberine was increased, while that of epiberberine and coptisine was decreased. The results indicated that the nonpolar substances existing in Zoagumhwan did not decrease the extraction yield of all alkaloids. The developed pre-processing method through removal of nonpolar substances in *Evodiae fructus* by SFE provided significant benefits in terms of extraction efficiency of the total alkaloids. Epiberberine, coptisine, palmatine and berberine all contain the –OCH_2_– group, which increases the dissolution rate under normal circumstance due to its polarity. However, with the absence of the role of the volatile oil when boiling, these four alkaloids in the solution vould reach new dissolution equilibrium. Epiberberin is the isomer of berberine, and would transfer into berberine for stability reasons. In the same way, the instability of –OCH_2_O– group would result in the decrease of content of coptisine. Thus, the above results were produced through a complicated interaction among chemicals, radicals, or ions in the extraction process. To confirm this deduction, further experiments should be conducted in the future.

## 3. Experimental

### 3.1. Materials and Reagents

*Coptidis rhizoma* and *Evodiae fruvtus * were purchased from Lv-Ye Pharm. Co. Ltd (Beijing, China) and authenticated by Professor Xiaohe Xiao (China Military Institute of Chinese Materia Medica, 302 Military Hospital of China). Columbamine hydrochloride (COL, No. 110711-200708), berberine hydrochloride (BER, No. 110717-200911), jateorrhizine hydrochloride (JAT, No. 110733-201108) and palmatine hydrochloride (PAL, No. 110732-201108) were supplied by the National Institute for the Control of Pharmaceutical and Biological Products in China. Coptisine hydrochloride (COP, No. 10/081025) and epiberberine sulphate (EPI, No. 10/081015) were supplied by Rong-He Pharm. Science and Technology Co. Ltd. (Shanghan, China). The purity of all reference compounds was more than 98.0%. Phosphoric acid was analytical purity from Beijing Chemical Works (Beijing, China). Methanol and acetonitrile were all of chromatographic purity from Fisher Chemicals (Pittsburgh, PA, USA). The water was double distilled water.

### 3.2. Instruments

A HA221-50-06 supercritical fluid extraction apparatus was purchased from Nantong Hua-An Supercritical Fluid Extraction Co. Ltd. (Nantong, Jiangsu, China). UPLC analysis was performed on a Waters Acquity Ultra performance liquid chromatography (UPLC) system (Waters Corp., Milford, MA, USA), equipped with a binary solvent delivery pump, an auto sampler and a photodiode array detector.

### 3.3. Chromatographic Conditions

The chromatographic separation of six alkaloids was performed on a Waters Acquity BEH C_18_ (50 mm × 2.1 mm, 1.7 μm) column. The mobile phase consisted of phosphoric acid aqueous solution and acetonitrile, both including 0.05% (*v/v*) buffer solution. The gradient elution procedure has been listed in Table 1. The detection wavelength was set at 270 nm. The injection volume was 1 μL while the column was maintained at 22.0 ± 0.5 °C.

### 3.4. SFE Conditions

The SFE conditions of Zhang *et al*. [10] were used with slight modifications. The flow rate (CO_2_) was kept at 4.7–15.7 L/h, extraction pressure was retained at 20–25 MPa, extraction time was 2 h, extraction temperature was maintained at 40.0 ± 2.0 °C and separation vessel II and vessel II were set 8 MPa and 6 MPa, respectively.

### 3.5. Preparation of Standard Solution

Mixed standard solution was prepared by dissolving six alkaloids, namely columbamine (7.8 mg), epiberberine (6.4 mg), jatrorrhizine (8.6 mg), coptisine (9.6 mg), palmatine (7.6 mg) and berberine (11.4 mg), with methanol (10 mL) in a flask. Then, the solution was transferred into a 50 mL volumetric flask, and made up to 50 mL. Finally, the standard stock solution was filtered through a 0.22 μm MILLEX GV syringe filter (Millipore, Bedford, MA, USA) prior to injection.

### 3.6. Pre-Processing of Crude Materials

#### 3.6.1. Pre-Processing by Direct Water Decoction

*Coptidis rhizoma* and *Evodiae fructus * were crushed to pieces and mixed together thoroughly at a ratio of 6:1 (w/w). Then, the mixture was macerated in warm water (40 °C) for 30 min and decocted with water three times, 2 h for the first time, 1 h for the second time and 0.5 h for the third time, respectively. The filtrates from each decoction were blended and concentrated to a thick solution using a rotary evaporator (BÜCHI, Flawil, Switzerland) at 75 °C. The concentrated sample was dried in a vacuum oven at 50 °C, and the percentage yield was 26.25%.

#### 3.6.2. Pre-Processing through Removal of Nonpolar Substances in Evodiae Fructus by SFE

Essential oil was removed firstly from *Evodiae fructus* by SFE under the above-described conditions. *Coptidis rhizoma* and *Evodiae fructus* without nonpolar substances at ratio of 6:1 (*w/w*) was mixed thoroughly and decocted with the same procedure as that of direct water decoction. The concentrated sample was dried in a vacuum oven at 50 °C, and the percentage yield was 25.72%.

#### 3.6.3. Pre-Processing through Removal of Nonpolar Substances in Zoagumhwan by SFE

*Coptidis rhizoma* and *Evodiae fructus* were mixed thoroughly at a ratio of 6:1 (*w/w*). Then, essential oil in the mixture was removed by SFE under the above-described conditions. Zoagumhwan without nonpolar substances was decocted with the same procedure as that of direct water decoction. The concentrated sample was dried in a vacuum oven at 50 °C, and the percentage yield was 27.54%.

#### 3.6.4. Pre-Processing through Removal of Nonpolar Substances in Zoagumhwan by Steam Distillation

*Coptidis rhizoma* and *Evodiae fructus* were thoroughly mixed first at a ratio of 6:1 (*w/w*). Then, the extraction method of steam distillation described in the Chinese Pharmacopoeia [16] was applied to remove nonpolar substances from the mixture. Zoagumhwan without essential oil was decocted with the same procedure as that of direct water decoction. The concentrated sample was dried in a vacuum oven at 50 °C, and the percentage yield was 24.58%.

### 3.7. Preparation of Zoagumhwan Sample Solutions

The concentrated and dried powders, namely ZGW I (78.75 mg), ZGW II (77.16 mg), ZGW III (82.62 mg) and ZGW IV (73.74 mg) from the above-described pre-processing methods of crude materials were dissolved in 10 mL methanol, shaken thoroughly and transferred into a flask. After being making up to 25 mL with methanol, the solutions were filtered through a 0.22 μm filter prior to UPLC analysis. For convenience, the sample solutions from the above-described four different pre-processing methods were labeled as ZGW I, II, III and IV, respectively.

### 3.8. Data Analysis

The Waters Empower software was used to collect and process the chromatograms data. Through importing the peak area and retention time in mean chromatograms, all parameters, including linear equation, correlations, and RSD, were calculated and analyzed using SPSS 13.0.

## 4. Conclusions

An easy and economical UPLC-PDA chromatographic procedure has been developed and successfully applied to determine the contents of six major alkaloids in Zoagumhwan extracts after different pre-processing technologies were used on the crude materials. The UPLC-PDA method was selective, sensitive and reproducible for the simultaneous determination of six alkaloids with low LOD and LOQ. The results have shown that the pro-processing technology through removal of nonpolar substances in *Evodiae fructus* by SFE could greatly improve the extraction efficiency of the main active components in Zoagumhwan. Thus, in the future, this described pro-processing method combined with UPLC analysis should be preferred and applied to pharmaceutical products of Zoagumhwan in order to improve the production technology, and to evaluate the productivity.
